# Corepressive function of nuclear receptor coactivator 2 in androgen receptor of prostate cancer cells treated with antiandrogen

**DOI:** 10.1186/s12885-016-2378-y

**Published:** 2016-05-25

**Authors:** Keisuke Takeda, Noboru Hara, Tsutomu Nishiyama, Masayuki Tasaki, Fumio Ishizaki, Yoshihiko Tomita

**Affiliations:** Division of Urology, Department of Regenerative and Transplant Medicine, Graduate School of Medical and Dental Sciences, Niigata University, Niigata, Japan; Division of Molecular Oncology, Department of Signal Transduction Research, Graduate School of Medical and Dental Sciences, Niigata University, Niigata, Japan; Asahimachi 1, Niigata, 951-8510 Japan

**Keywords:** Androgen receptor, Antiandrogen, Coactivator, Corepressor

## Abstract

**Background:**

Recruitment of cofactors in the interaction of the androgen receptor (AR) and AR ligands plays a critical role in determining androgenic/antiandrogenic effects of the AR ligand on signaling, but the functions of key cofactors, including nuclear receptor coactivator (NCOA), remain poorly understood in prostate cancer cells treated with AR ligands.

**Methods:**

We examined prostate cancer cell lines LNCaP and VCaP expressing mutated and wild-type ARs, respectively, to clarify the significance of NCOAs in the effect of antiandrogens. Hydroxyflutamide showed antagonistic activity against VCaP and an agonistic effect on LNCaP. Bicalutamide served as an antagonist for both. We analyzed mRNA transcription and protein expression of NCOAs in these cells pretreated with dihydrotestosterone and thereafter treated with the mentioned antiandrogens. Transcriptional silencing of candidate NCOAs and AR was performed using small interfering RNA (siRNA). Cell proliferation was evaluated with MTT assay.

**Results:**

LNCaP treated with bicalutamide showed an about four-fold increase in the expression of NCOA2 mRNA compared to those pretreated with dihydrotestosterone alone (*P* <0.01). In VCaP pretreated with dihydrotestosterone, transcriptions of NCOA2 and NCOA7 were slightly increased with bicalutamide (1.96- and 2.42-fold, respectively) and hydroxyflutamide (1.33-fold in both). With Western blotting, the expression of NCOA2 protein also increased in LNCaP cells treated with bicalutamide compared with that in control cells pretreated with dihydrotestosterone alone. Following silencing with siRNA for NCOA2, PSA levels in media with LNCaP receiving bicalutamide were elevated compared with those in non-silencing controls (101.6 ± 4.2 vs. 87.8 ± 1.4 ng/mL, respectively, *P* =0.0495). In LNCaP cells treated with dihydrotestosterone and bicalutamide, NCOA2-silencing was associated with a higher proliferation activity compared with non-silencing control and AR-silencing.

**Conclusion:**

NCOA2, which has been thought to be recruited as a coactivator, possibly plays a corepressive role in AR of prostate cancer cells when treated with antiandrogens, suggesting its potential as a therapeutic target.

**Electronic supplementary material:**

The online version of this article (doi:10.1186/s12885-016-2378-y) contains supplementary material, which is available to authorized users.

## Background

NR3C4 (nuclear receptor subfamily 3, group C, member 4), also well-known as the androgen receptor (AR), is a member of the nuclear hormone receptor superfamily of ligand-regulated transcription factors [1, 2]. It has been well-established that the androgen-AR interaction is involved in the proliferation of both benign and malignant prostate epithelial cells, and the role played by the androgen-AR interaction has been a therapeutic target [3, 4]. Androgen deprivation therapy (ADT) has thus been the mainstay for patients with metastatic prostate cancer and non-metastatic high-risk disease to prevent recurrence after definitive local therapy [5, 6].

Patients who have undergone ADT often show resistance to it, and develop castration-resistant prostate cancer (CRPC) [2, 7]. Despite being refractory to ADT, experimental studies as well as clinical practice suggest that CRPC has AR, which remains transcriptionally and functionally active beyond late-stage disease; human kallikrein (KLK) 3/prostate-specific antigen (PSA), whose production is regulated by androgen-dependent transcription, continues to elevate in the serum in men with CRPC receiving ADT [2–4, 8]. Such contradictions cannot be explained just by AR mutations or amplification [9]. Recently, the agonistic or antagonistic role of the AR ligand has been shown to be determined by the recruitment profile of cofactors in the AR complex, and it has also been suggested that the altered recruitment of cofactors in the androgen-AR interaction and the signaling pathway thereof are involved in the development of CRPC, and possibly have an impact on oncological outcomes [10–12]; however, relevant studies are limited.

Combined castration and peroral antiandrogens/AR-antagonists have been associated with better survival outcomes in men with prostate cancer compared with castration alone, although some studies suggest that their advantage may be limited in advanced disease [4, 5, 7]. Interestingly, antiandrogens represented by bicalutamide and hydroxyflutamide can give an agonistic effect; castrated men treated with antiandrogens, who have disease progression thereafter, occasionally show an decrease in serum PSA and improvement in disease following the discontinuation of them. This phenomenon of disease remission after the withdrawal of antiandrogens has been regarded as antiandrogen-withdrawal syndrome, and men showing such PSA reduction are associated with better oncological outcomes [13–15]. Correspondingly, a few exploratory studies reported that commonly used antiandrogens showed agonistic activity in cells with increased AR levels [16]; the altered arrangement or recruitment of coactivators and corepressors to the promoters of AR target genes may account for the mentioned antagonist-agonist conversion. Additionally, enhanced expressions of AR possibly intensify signaling from low levels of residual ligands, and change the normal response to antiandrogens, leading to resistance to them. However, roles played by cofactors in AR complex in prostate cancer cells remain unclear; approaching them may clarify the mechanism of castration resistance as well as antiandrogen withdrawal syndrome, potentially developing innovative therapy.

In the present study, we examined the expression profile of AR cofactors in prostate cancer cells under various hormonal conditions in the presence/absence of antiandrogens to clarify the significance of nuclear receptor coactivators (NCOAs) in the effect of antiandrogens, and to identify cofactors as targets for prostate cancer treatment.

## Methods

### Cells, agents, and antibodies

The protocol of this research project was approved by a suitably constituted Ethics Committee of Niigata University School of Medicine (#2050), and all experimental protocols for cell experimentation did not require ethical review and approval. Human prostate cancer cell lines LNCaP and VCaP were purchased from the American Type Culture Collection (Manassas, VA, USA). Hydroxyflutamide and bicalutamide, antagonists against AR, were purchased from Toronto Research Chemicals Inc. (Toronto, Canada) and Tocris Bioscience (Bristol, UK), respectively. Testosterone and dihydrotestosterone (DHT) were purchased from Steraloids (Wilton, NH, USA). Anti-NCOA2 (ab10491, Lot: GR167494-1) was obtained from Abcam plc. (CAMRIDGE, UK). Anti-beta actin (A5441, Lot: 122M478V) was obtained from SIGMA-ALDRICH Corp. (St. Louis, MO, USA).

### Cell culture

Cells were cultured in Roswell Park Memorial Institute-1640 (Gibco; Life Technologies, Carlsbad, CA, USA), supplemented with 10 % heat-inactivated fetal bovine serum (FBS), 1 % MEM nonessential amino acids, 1 % sodium pyruvate solution 100 mM, 0.14 % NaHCO_3,_ and 80 mg/L of kanamycin, at 37 °C in a humidified, 5 % CO_2_ atmosphere. These cells grown to subconfluence were switched to steroid hormone-depleted medium without phenol-red, containing 10 % charcoal-dextran stripped FBS (Biowest, Paris, France), and were then exposed to hydroxyflutamide or bicalutamide at 10^−5^ M for 3 days. For the Tandem-R PSA test (Beckman Coulter Inc., San Diego, CA, USA) in media, cells were plated at a population of 1 × 10^5^ cells/mL in triplicate.

### RNA extraction and quantification of gene expression by quantitative-PCR

We analyzed mRNA transcription levels of cofactors in LNCaP and VCaP cells pretreated with DHT of 10^−9^ M and thereafter treated with bicalutamide or hydroxyflutamide at 10^−5^ M. mRNA expressions of AR-related genes were determined by quantitative PCR. Cells were plated at a concentration of 5 × 10^5^ cells per 25-cm^2^ flasks (5 mL of media) (Falcon Labware, Lincoln Park, NJ, USA) for RNA isolation. Total RNA was isolated with Ambion’s RNAqueous-4PCR Kit (Applied Biosystems; Life Technologies) and cDNA was synthesized by reverse-transcription according to the protocol of the High-Capacity cDNA Reverse Transcription Kits (Applied Biosystems). The expression levels of genes and the internal reference beta-actin were estimated using quantitative PCR with the TaqMan system and ABI 7500 Sequence Detection System (Applied Biosystems). The detectors/probes were purchased from Applied Biosystems. Each experiment was triplicated, and we used the delta-delta Ct method for analysis. An increase with ratio values of 2.5-fold or higher in the transcription was considered indicative of significant overexpression [17, 18].

### Transcriptional silencing using small interfering RNA (siRNA)

LNCaP cells (passage number: 19 times) cultured in steroid hormone-depleted media containing 10 % charcoal-dextran stripped FBS were treated with 10^−9^ M DHT, and were prepared for the transfection of si-RNAs. A total of 5 × 10^5^ LNCaP cells were resuspended in 100 μL of buffer R with 5 × 10^−6^ M siRNA for NCOA2 or control non-silencing siRNA, and were transfected in 100 μL of Neon tip with the Neon transfection system (Life Technologies) using two pulses (1,100-V input pulse voltage/20-ms input pulse width). NCOA2-transfected cells were plated at a concentration of 1 × 10^5^ cells/mL per 25-cm^2^ flasks, and cultured in phenol-red free media with 10 % charcoal-dextran stripped of FBS with DHT of 10^−9^ for 3 days before the experiment. Functions of cofactors with small interfering RNA (siRNA)-based silencing were evaluated by measuring the concentration of PSA in cell culture media [19].

### Protein extract and Western blot assays

The cell tissues were prepared in lysis solution (PBS containing 1.0 % Triton-X 100 and 20 mM HEPES), and the supernatant was collected after being centrifuged at 25,000 × g for 30 min at 4 °C. Aliquots of proteins (24 μg of each sample) were prepared using sample buffer, heated for denaturing at 70 °C for 10 min, and separated on a 10 % Bis-Tris SDS-PAGE gel. Subsequently, they underwent electrophoresis and were transferred to PVDF membranes using iBlot Gel Transfer Stacks and the iBlot Dry Blotting System (Novex; Life Technologies). Membranes were preincubated for one hr at room temperature with blocking buffer (5 % skim milk and 0.1 % Tween20 in PBS), and incubated with the first antibody (NCOA2 [ab10491], 1:500 dilution) overnight at 4 °C. Thereafter, membranes were exposed to peroxidase-labeled second antibodies for 1 h at room temperature. Protein expressions were visualized using the ECL Prime System (GE Healthcare, Buckinghamshire, UK) and a cooled CCD camera system (AE-9300z, ATTO CORP, Tokyo, Japan).

### MTT assay

A total of 5 × 10^5^ LNCaP cells were resuspended in 10 μL of buffer R with 5 × 10^−6^ M siRNA for NCOA2, AR, or non-silencing control, and were transfected in 10 μL of Neon tip with the Neon transfection system (Life Technologies) using two pulses (1,100-V input pulse voltage/20-ms input pulse width). Cells were plated at a concentration of 5 × 10^5^ cells/mL per 96-well assay plate, and were cultured in phenol-red free media with 10 % charcoal-dextran stripped of FBS with DHT of 10^−9^. Twenty μl of Celltiter 96 AQueous One Solution Reagent (Promega, Fitchburg, WI, USA) were pipetted into wells containing 100 μl of culture medium, incubated the plate at 37 °C in a humidified, 5 % CO_2_ atmosphere, and subsequently, the absorbance at 490 nm was recorded using iMARK (BIO-RAD, Hercules, CA, USA). Assays were performed every 24 h for 3 days.

### Statistical analysis

The Kruskal-Wallis test was used to verify the significance in differences in expression level of mRNA and cell proliferation analysis with MTT assay. The Mann–Whitney U test was used to compare changes in paired parameters before and after siRNA. The test was two-sided and *p* < 0.05 was considered significant. All analyses were performed using SPSS version 15.0 J (SPSS Inc., Chicago, IL, USA) on a Windows-based computer.

## Results

### Effects of bicalutamide or hydroxyflutamide treatment on KLK3/PSA and AR transcription in prostate cancer cells pretreated with DHT

**Fig. 1:**
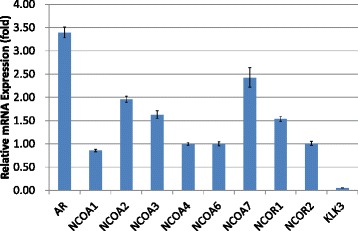
Alterations in transcription of androgen receptor (AR), NCOAs, NCORs, and human kallikrein 3/prostate-specific antigen (KLK3) in VCaP cells pretreated with dihydrotestosterone (DHT) and thereafter cultured with the addition of bicalutamide (BC). Relative mRNA expression levels were assessed in comparison with those in cells treated with DHT alone

**Fig. 2:**
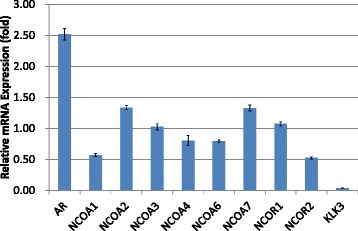
Transcriptional alterations of androgen receptor (AR), NCOAs, NCORs, and human kallikrein 3/prostate-specific antigen (KLK3) in VCaP cells pretreated with dihydrotestosterone (DHT) and subsequently cultured with the addition of hydroxyflutamide (HF). Relative mRNA expression levels were evaluated by comparing those in cells treated with DHT alone

**Fig. 3:**
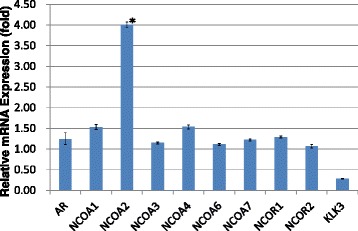
Alterations in transcription of androgen receptor (AR), NCOAs, NCORs, and human kallikrein 3/prostate-specific antigen (KLK3) in LNCaP cells pretreated with dihydrotestosterone (DHT) and thereafter cultured with the addition of bicalutamide (BC). Relative mRNA expression levels were assessed in comparison with those in cells treated with DHT alone. *P < 0.01 (Kruskal-Wallis test)

**Fig. 4:**
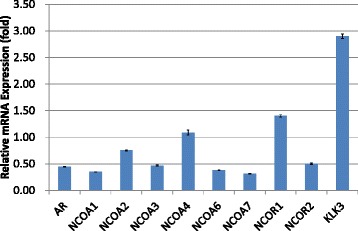
Transcriptional alterations of androgen receptor (AR), NCOAs, NCORs, and human kallikrein 3/prostate-specific antigen (KLK3) in LNCaP cells pretreated with dihydrotestosterone (DHT) and subsequently cultured with the addition of hydroxyflutamide (HF). Relative mRNA expression levels were evaluated in comparison with those in cells treated with DHT alone

In VCaP cells cultured in DHT-added media, the transcription level of KLK3/PSA with quantitative PCR was markedly reduced with bicalutamide treatment (0.05-fold, Fig. 1); this was also the case with hydroxyflutamide treatment (0.04-fold, Fig. 2). In LNCaP cells pretreated with DHT, KLK3/PSA transcription was downregulated with bicalutamide treatment (0.28-fold, Fig. 3), while hydroxyflutamide treatment clearly increased the transcription level of KLK3/PSA (2.90-fold, Fig. 4).

In VCaP cells cultured in DHT-added media, bicalutamide and hydroxyflutamide treatment significantly upregulated the transcription of AR (3.39- and 2.51-fold, respectively) (Figs. 1 and 2). In LNCaP cells pretreated with DHT, bicalutamide treatment slightly increased AR transcription (1.24-fold, Fig. 3), whereas AR transcription decreased with hydroxyflutamide (0.44-fold, Fig. 4).

### Impact of bicalutamide or hydroxyflutamide treatment on the transcription of NCOA and nuclear receptor corepressor (NCOR) families

Transcriptions of NCOA2 and NCOA7 were also elevated with bicalutamide (1.96- and 2.42-fold, respectively, Fig. 1) and hydroxyflutamide (1.33-fold in both, Fig. 2) in VCaP cells pretreated with DHT. LNCaP cells pretreated with DHT receiving bicalutamide showed a 4-fold increase in the expression of NCOA2 mRNA compared with those cultured with DHT pretreatment alone (Fig. 3); transcription levels of other coactivators such as NCOA1, NCOA3, and NCOA4 did not increase with bicalutamide. Also, corepressors such as NCOR1 and NCOR2 did not show an increase in the transcription level.

### Influence of knock-down of NCOA2 in LNCaP cells on the production of KLK3/PSA

**Fig. 5:**
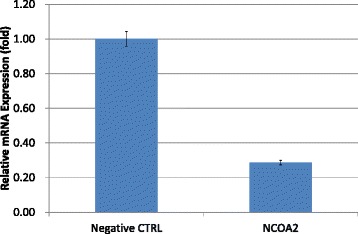
The efficacy of siRNA for silencing NCOA2 in transcriptions. 1.00 ± 0.04 (control) vs. 0.29 ± 0.01, *P* = 0.0495

**Fig. 6:**
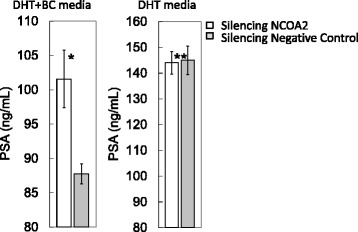
Impact of knock-down of NCOA2 using siRNA on the production of prostate-specific antigen (PSA) in LNCaP cells cultured with dihydrotestosterone (DHT) plus bicalutamide (BC) (left columns) and those treated with DHT alone. *101.6 ± 4.2 vs. 87.8 ± 1.4 ng/mL, respectively, *P* = 0.0495, **144 ± 4.4 vs. 145 ± 5.6 ng/mL, respectively, n.s

We also established knock-down models of NCOA2 in LNCaP cells with transcriptional silencing using small interfering RNA (siRNA). Transcription levels of NCOA2 were significantly reduced compared with negative controls (Fig. 5). Thereafter, knock-down of NCOA2 with siRNA showing most efficient silencing was performed in LNCaP cells pretreated with DHT; the KLK3/PSA concentration in media increased compared with that in non-silencing controls (Fig. 6, *P* =0.0495).

### Protein expressions of NCOA2 in LNCaP cells cultured with DHT and bicalutamide

**Fig. 7:**
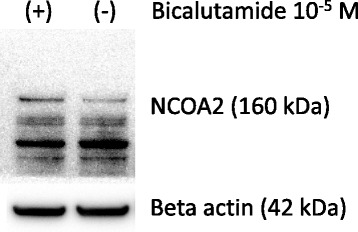
Protein expression levels of NCOA2 with Western Blot in LNCaP cells cultured with dihydrotestosterone (DHT) plus bicalutamide (BC). The protein level of NCOA2 increased with compared to that without BC

With Western blotting, LNCaP cells pretreated with DHT showed an increased protein level of NCOA2 with bicalutamide than those without (Fig. 7).

### Alterations in cell proliferation of LNCaP with silencing of NCOA2 or AR

**Fig. 8:**
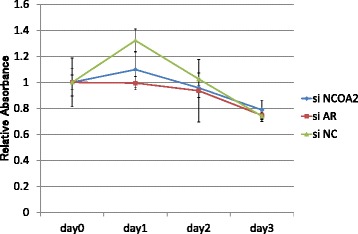
Relative absorbance with MTT assay in LNCaP cells cultured with dihydrotestosterone. There was no difference among proliferations in the NCOA2-silencing, AR-silencing, and non-silencing cells

**Fig. 9:**
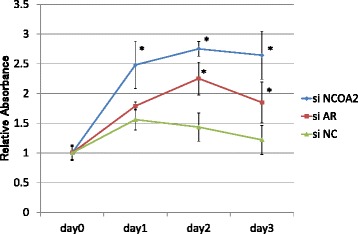
Relative absorbance with MTT assay in LNCaP cells cultured with dihydrotestosterone (DHT) plus bicalutamide (BC). NCOA2-silencing cells showed an increased proliferation compared with non-silencing control cells and those with silencing androgen receptor (AR). LNCaP cells with silencing AR showed decreased proliferation compared with those silencing NCOA2. *P < 0.01 (Kruskal-Wallis test)

There was no difference among proliferations in the NCOA2-silencing, AR-silencing, and non-silencing cells cultured with dihydrotestosterone alone (Fig. 8). Relative absorbance with MTT assay in LNCaP cells cultured with DHT plus bicalutamide was shown in Fig. 9. Cells with NCOA2-silencing showed a higher proliferation activity compared with non-silencing control cells and those with AR-silencing.

## Discussion

LNCaP and VCaP cells have mutated and wild-type AR, respectively [20, 21]. Hydroxyflutamide has an agonistic effect on LNCaP cells, and bicalutamide serves as an antagonist against them [22, 23]. For VCaP cells, the effect of these antiandrogens has not been determined. In the current study, both bicalutamide and hydroxyflutamide treatments reduced KLK3/PSA transcription in VCaP cells (Figs. 1 and 2), while the former decreased and the latter increased KLK3/PSA transcription in LNCaP (Figs. 3 and 4), suggesting the different nature of AR in the response to antiandrogens between the two cell types. The transcriptional regulation in AR in response to antiandrogens also differed between VCaP and LNCaP cells (Figs. 1, 2, 3 and 4), but bicalutamide inhibited KLK3/PSA transcription in both VCaP and LNCaP cells to a similar extent.

The present study verified that the absence of DHT negated the role of antiandrogens; transcriptions of AR, NCOAs, and KLK3/PSA were not altered by antiandrogens in the androgen-deprived milieu, except for KLK3/PSA being increased by hydroxyflutamide. In the absence of endogenous ligands, AR is segregated in the cytoplasm, with its nuclear localization sequence (NLS) masked by heat-shock proteins. Binding to ligands and the separation of these chaperones cause AR to dimerize, and lead to conformational changes and the exposure of the NLS [24–27]. Thus, the nuclear translocation of AR and binding to androgen response elements (AREs) activate androgen-responsive genes. This process is transcriptionally regulated and post-transcriptionally modified through various mechanisms involving interactions with multiple coactivator and corepressor proteins [28–30]. AR activity is modulated by the recruitment of multitudes of positive and negative cofactors, being closely associated with the regulation of protein stability, interaction with others, intracellular receptor localization, and alteration of the AR structure. Thus, the elucidation of cofactor-related modifications of AR may be a promising approach to develop novel and efficient therapeutic options.

In our study, the transcription level of NCOA2 increased with bicalutamide treatment in LNCaP cells (Figs. 3 and 7); transcription levels of other coactivators were not altered. On the other hand, such upregulation of NCOA2 was not marked in VCaP, and the difference may possibly be due to the aforementioned issues in AR and the response to antiandrogens being different between the 2 types of cell. Moreover, knock-down of NCOA2 in LNCaP cells increased the KLK3/PSA concentration in media (Figs. 5 and 6), and cell proliferation analysis with MTT assay further supported the mentioned novel finding (Figs. 8 and 9); NCOA2 is possibly associated with the downregulation of AR signaling in prostate cancer cells treated with bicalutamide. In the presence of bicalutamide, interestingly, knock-down of NCOA2 was associated with a higher cell proliferation than knock-down of AR. These results suggest that NCOA2 plays an inhibitory role in prostate cancer cells treated with bicalutamide. Steroid receptor coactivators represented by NCOA2 and NCOA3 are key regulators, having multiple effects, of transcription factors necessary for cancer cell proliferation, survival, and metastasis [31]. Their overexpression and/or overactivation has been shown in a number of human cancers with various genomic, transcriptional, and posttranslational mechanisms, and are associated with refractory disease leading to poor outcomes [31]. In human prostate cancer, NCOAs have been reported to regulate cell proliferation and invasion and be involved in castration resistance, coupled with AR transcriptional activity [32–34]. NCOA2 has therefore been shown to play a role as a coactivator in the androgen-AR interaction [35–38]; however, there has been no study examining the function of NCOAs in prostate cancer cells treated with antiandrogens. The current results suggest that NCOA2 may possibly explain the reduction of PSA on the withdrawal or conversion of antiandrogens; it possibly also serves as a corepressor in the presence of antiandrogens in prostate cancer cells pretreated with androgens. It is thus necessary to characterize the functional and recruitment profile of each cofactor-AR complex and signaling in prostate cancer in accordance with antagonists as well as agonists.

The present study had several limitations. With the current cell lines and hormonal milieu, cell proliferation assays lack in reproducibility. Knock-down studies with siRNA require further verification of the response, and the application of different siRNAs is necessary to rule out unrelated effects.

## Conclusions

Although the recruitment of NCOA2 has been thought to induce agonistic signaling in AR, the current study showed that it possibly also serves as a corepressor in the presence of antiandrogens in prostate cancer cells cultured in a physiological hormonal milieu, suggesting its potential as a therapeutic target for prostate cancer. Characterization of the function and recruitment profile of each cofactor related to AR signaling brought about by various AR ligands may lead to advanced therapy for men with prostate cancer.

## Abbreviations

ADT, androgen deprivation therapy; AR, androgen receptor; ARE, androgen response element; CRPC, castration-resistant prostate cancer; DHT, dihydrotestosterone; FBS, fetal bovine serum; KLK, human kallikrein; NCOA, nuclear receptor coactivator; NCOR, nuclear receptor corepressor; PSA, prostate-specific antigen; siRNA, small interfering RNA

## Additional files

Additional file 1: Figure S1. Screening of the siRNA efficacy on target mRNA expression levels. Catalog numbers were s20580, s20581, and s20582 (Neon Transfection System; Life Technologies, Carlsbad, CA, USA) for siRNA NCOA2 #1 (siNCOA2 #1), #2 (siNCOA2 #2), and #3 (siNCOA2 #3), respectively. Based on this result, siRNA NCOA2 #2 was selected, and the relevant data were indicated in Fig. 5. (DOC 54 kb) Additional file 2: Table S1. Ct values of gene expression assessed by quantitative PCR in VCaP cells cultured with dihydrotestosterone-added media. Delta-delta Ct value was calculated from the following formula: delta(target gene Ct – internal control Ct) with specific cell culture media - delta(target gene Ct – internal control Ct) with standard cell culture media. (DOC 32 kb) Additional file 3: Table S2. Ct values of quantitative PCR in VCaP cells cultured with dihydrotestosterone- and bicalutamide-added media. (DOC 31 kb) Additional file 4: Table S3. Ct values of quantitative PCR in VCaP cells cultured with dihydrotestosterone- and hydroxyflutamide-added media. (DOC 31 kb) Additional file 5: Table S4. Ct values of quantitative PCR in LNCaP cells cultured with dihydrotestosterone-added media. (DOC 31 kb) Additional file 6: Table S5. Ct values of quantitative PCR in LNCaP cells cultured with dihydrotestosterone- and bicalutamide-added media. (DOC 31 kb) Additional file 7: Table S6. Ct values of quantitative PCR in LNCaP cells cultured with dihydrotestosterone- and hydroxyflutamide-added media. (DOC 31 kb) Additional file 8: Figure S2. Full membranes of Western blotting (corresponding to Fig. 7). The membrane in the left panel showed NCOA2 staining part, and the right showed actin beta staining part. Starting from the left lane, NCOA2 expressions in LNCaP with bicalutamide and without bicalutamide, and positive control were shown. Twenty-seven ug of Jurkat cell lysate (Catalog #611451, BD Biosciences, Franklin Lakes, NJ, USA) was used as positive control. Densitometry data of Western blotting were attached below them. (DOC 1266 kb)
