# Supplementary material for: Corepressive function of nuclear receptor coactivator 2 in androgen receptor of prostate cancer cells treated with antiandrogen
Source: BMC Cancer. 2016 May 25;16:332. doi: 10.1186/s12885-016-2378-y (PMC4880970; doi:10.1186/s12885-016-2378-y)
Supplement: Additional file 1: Figure S1. — Screening of the siRNA efficacy on target mRNA expression levels. Catalog numbers were s20580, s20581, and s20582 (Neon Transfection System; Life Technologies, Carlsbad, CA, USA) for siRNA NCOA2 #1 (siNCOA2 #1), #2 (siNCOA2 #2), and #3 (siNCOA2 #3), respectively. Based on this result, siRNA NCOA2 #2 was selected, and the relevant data were indicated in Fig. 5. (DOC 54 kb) [file 12885_2016_2378_MOESM1_ESM.doc]

**Additional file 1: Figure S1**

Screening of the siRNA efficacy on target mRNA expression levels. Catalog numbers were s20580, s20581, and s20582 (Neon Transfection System; Life Technologies, Carlsbad, CA, USA) for siRNA NCOA2 #1 (siNCOA2 #1), #2 (siNCOA2 #2), and #3 (siNCOA2 #3), respectively. Based on this result, siRNA NCOA2 #2 was selected, and the relevant data were indicated in Figure 5.

**
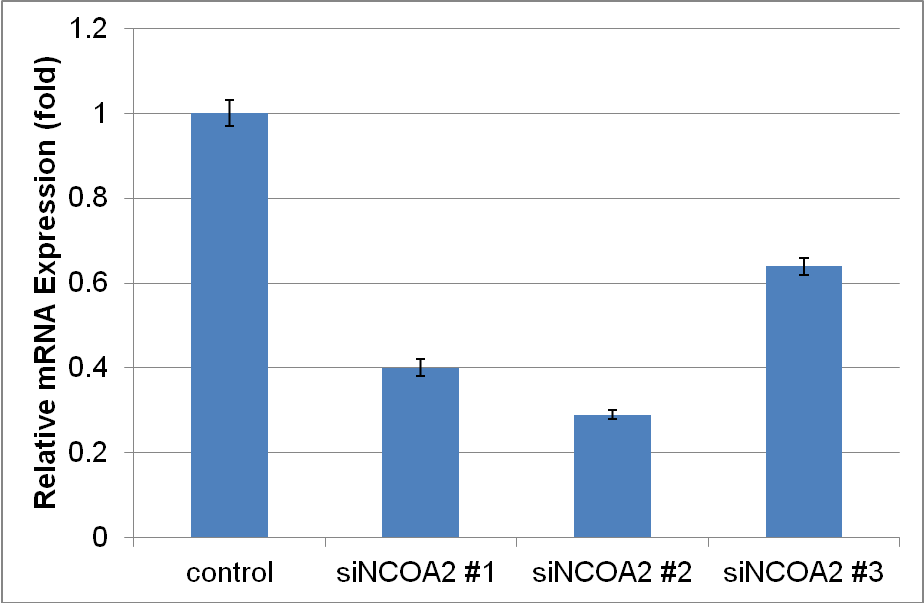
**
