# Supplementary material for: Corepressive function of nuclear receptor coactivator 2 in androgen receptor of prostate cancer cells treated with antiandrogen
Source: BMC Cancer. 2016 May 25;16:332. doi: 10.1186/s12885-016-2378-y (PMC4880970; doi:10.1186/s12885-016-2378-y)
Supplement: Additional file 2: Table S1. — Ct values of gene expression assessed by quantitative PCR in VCaP cells cultured with dihydrotestosterone-added media. Delta-delta Ct value was calculated from the following formula: delta(target gene Ct – internal control Ct) with specific cell culture media - delta(target gene Ct – internal control Ct) with standard cell culture media. (DOC 32 kb) [file 12885_2016_2378_MOESM2_ESM.doc]

**Additional file 2: Table S1**

Ct values of gene expression assessed by quantitative PCR in VCaP cells cultured with dihydrotestosterone-added media. Delta-delta Ct value was calculated from the following formula: delta(target gene Ct – internal control Ct) with specific cell culture media - delta(target gene Ct – internal control Ct) with standard cell culture media.

| **Detector** | **Avg Ct** | **Avg dCt** | **dCt Std Err** |
| --- | --- | --- | --- |
| **AR** | **24.269** | **2.071** | **0.055** |
| **NCOA1** | **26.06** | **3.861** | **0.035** |
| **NCOA2** | **26.253** | **4.055** | **0.051** |
| **NCOA3** | **27.003** | **4.805** | **0.034** |
| **NCOA4** | **33.012** | **10.813** | **0.125** |
| **NCOA6** | **27.776** | **5.578** | **0.046** |
| **NCOA7** | **29.552** | **7.353** | **0.037** |
| **NCOR1** | **26.275** | **4.077** | **0.052** |
| **NCOR2** | **25.736** | **3.537** | **0.044** |
| **KLK3** | **22.648** | **0.449** | **0.105** |
| **ACTB** | **22.198** |  |  |
