# Supplementary material for: Corepressive function of nuclear receptor coactivator 2 in androgen receptor of prostate cancer cells treated with antiandrogen
Source: BMC Cancer. 2016 May 25;16:332. doi: 10.1186/s12885-016-2378-y (PMC4880970; doi:10.1186/s12885-016-2378-y)
Supplement: Additional file 3: Table S2. — Ct values of quantitative PCR in VCaP cells cultured with dihydrotestosterone- and bicalutamide-added media. (DOC 31 kb) [file 12885_2016_2378_MOESM3_ESM.doc]

**Additional file 3: Table S2**

Ct values of quantitative PCR in VCaP cells cultured with dihydrotestosterone- and bicalutamide-added media.

| **Detector** | **Avg Ct** | **Avg dCt** | **dCt Std Err** |
| --- | --- | --- | --- |
| **AR** | **23.392** | **0.309** | **0.049** |
| **NCOA1** | **27.168** | **4.084** | **0.044** |
| **NCOA2** | **26.168** | **3.085** | **0.048** |
| **NCOA3** | **27.187** | **4.104** | **0.073** |
| **NCOA4** | **33.898** | **10.814** | **0.04** |
| **NCOA6** | **28.666** | **5.583** | **0.063** |
| **NCOA7** | **29.163** | **6.08** | **0.126** |
| **NCOR1** | **26.54** | **3.457** | **0.047** |
| **NCOR2** | **26.607** | **3.523** | **0.063** |
| **KLK3** | **27.856** | **4.772** | **0.079** |
| **ACTB** | **23.083** |  |  |
