# Supplementary material for: Corepressive function of nuclear receptor coactivator 2 in androgen receptor of prostate cancer cells treated with antiandrogen
Source: BMC Cancer. 2016 May 25;16:332. doi: 10.1186/s12885-016-2378-y (PMC4880970; doi:10.1186/s12885-016-2378-y)
Supplement: Additional file 4: Table S3. — Ct values of quantitative PCR in VCaP cells cultured with dihydrotestosterone- and hydroxyflutamide-added media. (DOC 31 kb) [file 12885_2016_2378_MOESM4_ESM.doc]

**Additional file 4: Table S3**

Ct values of quantitative PCR in VCaP cells cultured with dihydrotestosterone- and hydroxyflutamide-added media.

| **Detector** | **Avg Ct** | **Avg dCt** | **dCt Std Err** |
| --- | --- | --- | --- |
| **AR** | **22.845** | **0.741** | **0.053** |
| **NCOA1** | **26.778** | **4.673** | **0.059** |
| **NCOA2** | **25.744** | **3.639** | **0.034** |
| **NCOA3** | **26.875** | **4.77** | **0.07** |
| **NCOA4** | **33.24** | **11.136** | **0.147** |
| **NCOA6** | **28.025** | **5.92** | **0.039** |
| **NCOA7** | **29.049** | **6.944** | **0.054** |
| **NCOR1** | **26.082** | **3.977** | **0.043** |
| **NCOR2** | **26.574** | **4.47** | **0.051** |
| **KLK3** | **27.345** | **5.24** | **0.046** |
| **ACTB** | **22.105** |  |  |
