# Supplementary material for: Corepressive function of nuclear receptor coactivator 2 in androgen receptor of prostate cancer cells treated with antiandrogen
Source: BMC Cancer. 2016 May 25;16:332. doi: 10.1186/s12885-016-2378-y (PMC4880970; doi:10.1186/s12885-016-2378-y)
Supplement: Additional file 5: Table S4. — Ct values of quantitative PCR in LNCaP cells cultured with dihydrotestosterone-added media. (DOC 31 kb) [file 12885_2016_2378_MOESM5_ESM.doc]

**Additional file 5: Table S4**

Ct values of quantitative PCR in LNCaP cells cultured with dihydrotestosterone-added media.

| **Detector** | **Avg Ct** | **Avg dCt** | **dCt Std Err** |
| --- | --- | --- | --- |
| **AR** | **23.882** | **3.05** | **0.072** |
| **NCOA1** | **25.121** | **4.288** | **0.067** |
| **NCOA2** | **27.309** | **6.476** | **0.046** |
| **NCOA3** | **27.154** | **6.322** | **0.043** |
| **NCOA4** | **31.041** | **10.209** | **0.046** |
| **NCOA6** | **27.287** | **6.454** | **0.048** |
| **NCOA7** | **25.324** | **4.491** | **0.042** |
| **NCOR1** | **26.623** | **5.791** | **0.045** |
| **NCOR2** | **26.452** | **5.619** | **0.05** |
| **KLK3** | **22.216** | **1.384** | **0.043** |
| **ACTB** | **20.833** |  |  |
