# Supplementary material for: Corepressive function of nuclear receptor coactivator 2 in androgen receptor of prostate cancer cells treated with antiandrogen
Source: BMC Cancer. 2016 May 25;16:332. doi: 10.1186/s12885-016-2378-y (PMC4880970; doi:10.1186/s12885-016-2378-y)
Supplement: Additional file 6: Table S5. — Ct values of quantitative PCR in LNCaP cells cultured with dihydrotestosterone- and bicalutamide-added media. (DOC 31 kb) [file 12885_2016_2378_MOESM6_ESM.doc]

**Additional file 6: Table S5**

Ct values of quantitative PCR in LNCaP cells cultured with dihydrotestosterone- and bicalutamide-added media.

| **Detector** | **Avg Ct** | **Avg dCt** | **dCt Std Err** |
| --- | --- | --- | --- |
| **AR** | **24.317** | **2.736** | **0.167** |
| **NCOA1** | **25.255** | **3.673** | **0.057** |
| **NCOA2** | **26.055** | **4.474** | **0.026** |
| **NCOA3** | **27.705** | **6.123** | **0.032** |
| **NCOA4** | **31.172** | **9.591** | **0.044** |
| **NCOA6** | **27.882** | **6.301** | **0.03** |
| **NCOA7** | **25.786** | **4.204** | **0.027** |
| **NCOR1** | **27.007** | **5.426** | **0.027** |
| **NCOR2** | **27.107** | **5.526** | **0.056** |
| **KLK3** | **24.782** | **3.201** | **0.029** |
| **ACTB** | **21.582** |  |  |
