# Supplementary material for: Corepressive function of nuclear receptor coactivator 2 in androgen receptor of prostate cancer cells treated with antiandrogen
Source: BMC Cancer. 2016 May 25;16:332. doi: 10.1186/s12885-016-2378-y (PMC4880970; doi:10.1186/s12885-016-2378-y)
Supplement: Additional file 7: Table S6. — Ct values of quantitative PCR in LNCaP cells cultured with dihydrotestosterone- and hydroxyflutamide-added media. (DOC 31 kb) [file 12885_2016_2378_MOESM7_ESM.doc]

**Additional file 7: Table S6**

Ct values of quantitative PCR in LNCaP cells cultured with dihydrotestosterone- and hydroxyflutamide-added media.

| **Detector** | **Avg Ct** | **Avg dCt** | **dCt Std Err** |
| --- | --- | --- | --- |
| **AR** | **24.437** | **4.227** | **0.021** |
| **NCOA1** | **26.026** | **5.816** | **0.019** |
| **NCOA2** | **27.097** | **6.887** | **0.02** |
| **NCOA3** | **27.638** | **7.428** | **0.034** |
| **NCOA4** | **30.298** | **10.088** | **0.059** |
| **NCOA6** | **28.064** | **7.854** | **0.029** |
| **NCOA7** | **26.395** | **6.185** | **0.027** |
| **NCOR1** | **25.512** | **5.302** | **0.02** |
| **NCOR2** | **26.827** | **6.618** | **0.041** |
| **KLK3** | **20.057** | **-0.152** | **0.022** |
| **ACTB** | **20.21** |  |  |
