# Supplementary material for: Corepressive function of nuclear receptor coactivator 2 in androgen receptor of prostate cancer cells treated with antiandrogen
Source: BMC Cancer. 2016 May 25;16:332. doi: 10.1186/s12885-016-2378-y (PMC4880970; doi:10.1186/s12885-016-2378-y)
Supplement: Additional file 8: Figure S2. — Full membranes of Western blotting (corresponding to Fig. 7). The membrane in the left panel showed NCOA2 staining part, and the right showed actin beta staining part. Starting from the left lane, NCOA2 expressions in LNCaP with bicalutamide and without bicalutamide, and positive control were shown. Twenty-seven ug of Jurkat cell lysate (Catalog #611451, BD Biosciences, Franklin Lakes, NJ, USA) was used as positive control. Densitometry data of Western blotting were attached below them. (DOC 1266 kb) [file 12885_2016_2378_MOESM8_ESM.doc]

**Additional file 8: Figure S2**

Full membranes of Western blotting (corresponding to Figure 7). The membrane in the left panel showed NCOA2 staining part, and the right showed actin beta staining part. Starting from the left lane, NCOA2 expressions in LNCaP with bicalutamide and without bicalutamide, and positive control were shown. Twenty-seven ug of Jurkat cell lysate (Catalog #611451, BD Biosciences, Franklin Lakes, NJ, USA) was used as positive control. Densitometry data of Western blotting were attached below them.

**
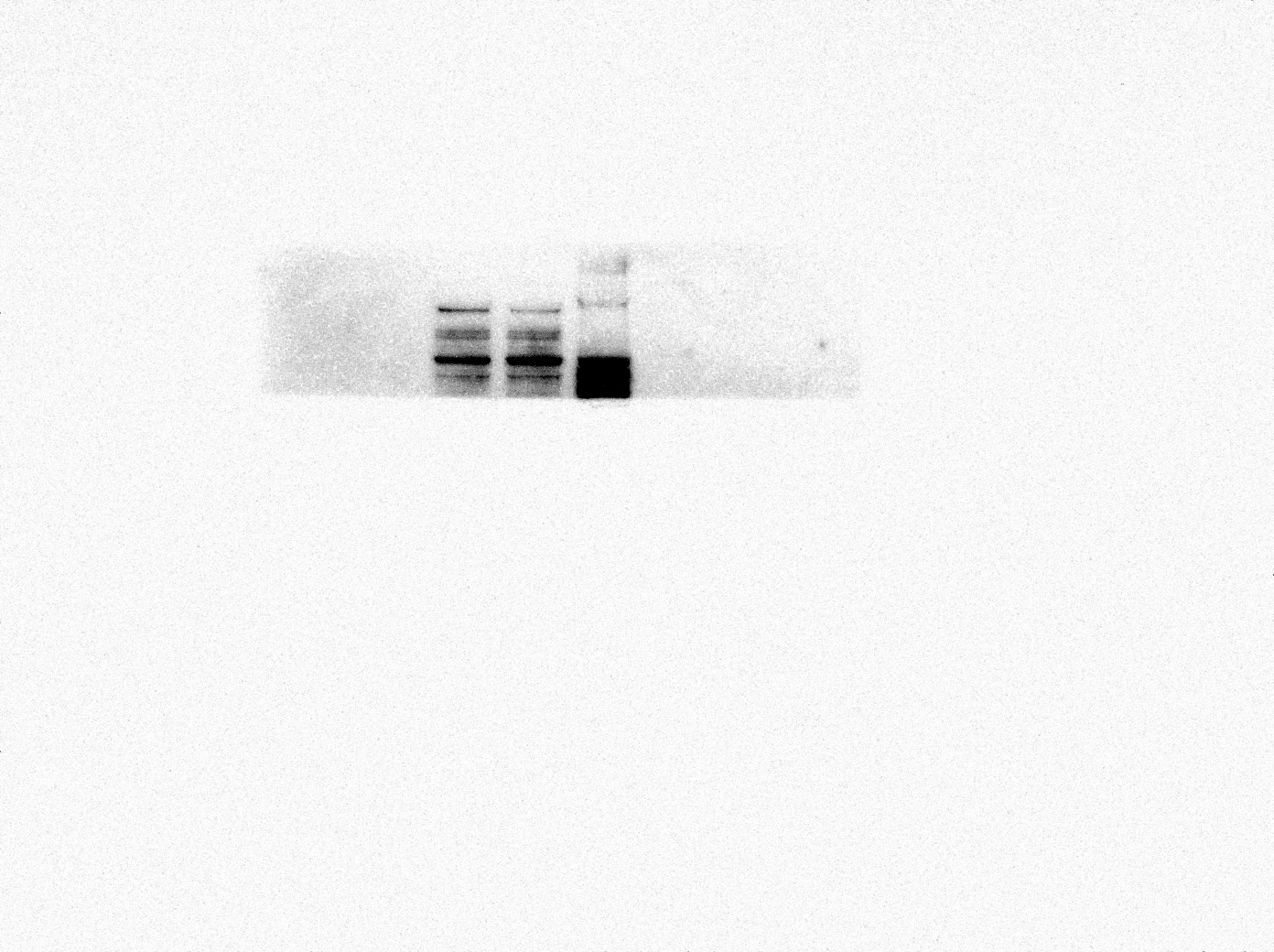

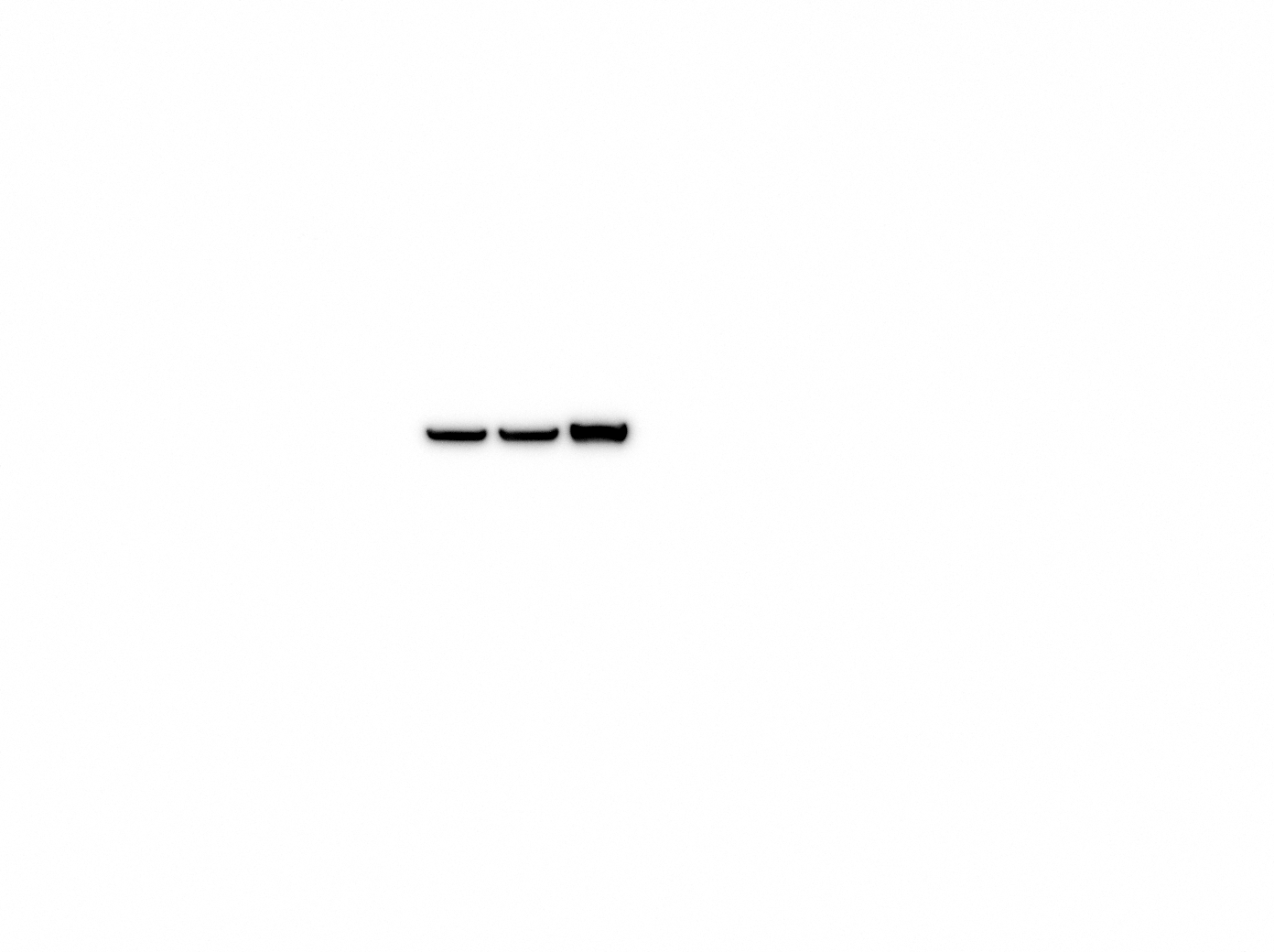
**

**bicalutamide + bicalutamide -**

**NCOA2 240406 33787**

**actin beta 6962836 7778404**
